# (Nitrato-κ*O*){*N*,*N*,*N*′,*N*′-tetra­kis­[(1*H*-benzimidazol-2-yl-κ*N* ^3^)meth­yl]cyclo­hexane-1,2-diamine}­lead(II) hemiaqua­{*N*,*N*,*N*′,*N*′-tetra­kis­[(1*H*-benzimidazol-2-yl-κ*N* ^3^)meth­yl]cyclo­hexane-1,2-diamine}­lead(II) trinitrate dihydrate

**DOI:** 10.1107/S1600536811050082

**Published:** 2011-11-30

**Authors:** Zuo-an Xiao, Ting-ting Jiang

**Affiliations:** a School of Chemical Engineering and Food Science, Xiangfan University, Xiangfan 441053, People’s Republic of China

## Abstract

In the title compound, [Pb(NO_3_)(C_38_H_38_N_10_)][Pb(C_38_H_38_N_10_)(H_2_O)_0.5_](NO_3_)_3_·2H_2_O, both Pb^II^ ions are coordinated in a distorted trigonal–prismatic environment by a hexa­dentate *N*,*N*,*N′*,*N′*-tetra­kis­[(1*H*-benzimidazol-2-yl)meth­yl]cyclo­hex­ane-1,2-diamine ligand. A nitrate and a half-occupancy water ligand form long coordination bonds to the Pb^II^ ions capping the trigonal–prismatic environment. In the crystal, the components are linked by N—H⋯O and O—H⋯O hydrogen bonds, forming a three-dimensional network. C—H⋯O inter­actions also occur.

## Related literature

For background to lead(II) complexes, see: Bazzicalupi *et al.* (1999 ▶); Kavallieratos *et al.* (2005 ▶); Schwerdtheger *et al.* (1992 ▶); Byriel *et al.* (1992 ▶). For a related structure, see: Zhang *et al.* (2007 ▶). For the synthesis of *N*,*N*,*N*′,*N*′-tetra­kis­(2-benz­imid­azol­ylmeth­yl)cyclo­hexane-1,2-diamine, see: Hendriks *et al.* (1982 ▶).
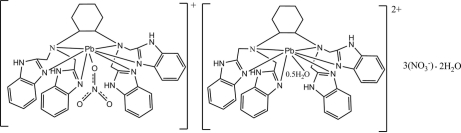


## Experimental

### 

#### Crystal data

[Pb(NO_3_)(C_38_H_38_N_10_)][Pb(C_38_H_38_N_10_)(H_2_O)_0.5_](NO_3_)_3_·2H_2_O
*M* *_r_* = 1978.0
Triclinic, 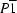

*a* = 12.5485 (8) Å
*b* = 18.5378 (11) Å
*c* = 19.2839 (12) Å
α = 64.516 (1)°
β = 81.957 (2)°
γ = 79.675 (1)°
*V* = 3973.9 (4) Å^3^
*Z* = 2
Mo *K*α radiation
μ = 4.31 mm^−1^
*T* = 298 K
0.20 × 0.10 × 0.10 mm

#### Data collection

Bruker SMART CCD diffractometer
Absorption correction: multi-scan (*SADABS*; Sheldrick, 1996 ▶) *T*_min_ = 0.479, *T*_max_ = 0.672
41656 measured reflections
15546 independent reflections
11020 reflections with *I* > 2σ(*I*)
*R*_int_ = 0.054

#### Refinement

*R*[*F* ^2^ > 2σ(*F* ^2^)] = 0.045
*wR*(*F* ^2^) = 0.101
*S* = 0.96
15546 reflections
1051 parameters
H-atom parameters constrained
Δρ_max_ = 1.81 e Å^−3^
Δρ_min_ = −0.54 e Å^−3^

### 

Data collection: *SMART* (Bruker, 2001 ▶); cell refinement: *SAINT* (Bruker, 2001 ▶); data reduction: *SAINT*; program(s) used to solve structure: *SHELXS97* (Sheldrick, 2008 ▶); program(s) used to refine structure: *SHELXL97* (Sheldrick, 2008 ▶); molecular graphics: *PLATON* (Spek, 2009 ▶); software used to prepare material for publication: *SHELXTL* (Sheldrick, 2008 ▶).

## Supplementary Material

Crystal structure: contains datablock(s) global, I. DOI: 10.1107/S1600536811050082/lh5344sup1.cif

Structure factors: contains datablock(s) I. DOI: 10.1107/S1600536811050082/lh5344Isup2.hkl

Additional supplementary materials:  crystallographic information; 3D view; checkCIF report

## Figures and Tables

**Table 1:** Hydrogen-bond geometry (Å, °)

*D*—H⋯*A*	*D*—H	H⋯*A*	*D*⋯*A*	*D*—H⋯*A*
N4*A*—H4*A*⋯O9^i^	0.86	2.14	2.926 (8)	151
N4*A*—H4*A*⋯O7^i^	0.86	2.45	3.242 (10)	153
N6*A*—H6*A*⋯O3	0.86	2.04	2.848 (7)	157
N8*A*—H8*A*⋯O11^ii^	0.86	2.12	2.898 (8)	150
N8*A*—H8*A*⋯O12^ii^	0.86	2.37	3.091 (8)	141
N10*A*—H10*D*⋯O2^iii^	0.86	2.03	2.890 (7)	175
N4*B*—H4*B*⋯O4^iv^	0.86	2.03	2.879 (6)	169
N4*B*—H4*B*⋯O6^iv^	0.86	2.57	3.256 (7)	138
N6*B*—H6*B*⋯O14	0.86	1.95	2.784 (6)	164
N8*B*—H8*B*⋯O13^v^	0.86	1.99	2.843 (6)	169
N10*B*—H10*C*⋯O5	0.86	2.06	2.852 (6)	152
O1*A*—H1*O*⋯O13	0.84	2.13	2.646 (13)	120
O1*A*—H2*O*⋯O10	0.84	1.79	2.630 (15)	178
O13—H3*O*⋯O10	0.84	2.46	3.126 (9)	137
O13—H4*O*⋯O1^v^	0.84	2.31	3.029 (8)	143
O14—H5*O*⋯O9^vi^	0.84	2.01	2.853 (8)	179
O14—H6*O*⋯O12^vii^	0.84	2.10	2.939 (8)	179
C13*B*—H13*B*⋯O7	0.93	2.50	3.312 (10)	146
C15*A*—H15*A*⋯O4	0.97	2.59	3.447 (7)	148
C18*B*—H18*B*⋯O10^vii^	0.93	2.45	3.377 (9)	171
C7*B*—H7*B*1⋯O6^iv^	0.97	2.31	3.227 (8)	157
C29*B*—H29*B*⋯O8	0.93	2.57	3.363 (10)	144
C31*A*—H31*B*⋯O1^iii^	0.97	2.41	3.313 (8)	155

Symmetry codes: (i) 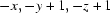; (ii) 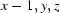; (iii) 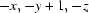; (iv) 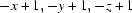; (v) 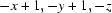; (vi) 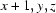; (vii) 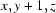.

**Fig. 1.**
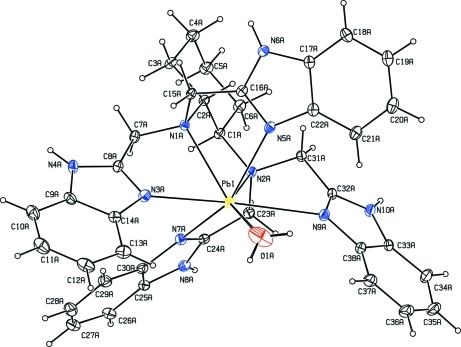
A view of the molecular structure of the Pb1 containing cation with displacement ellipsoids drawn at the 10% probability level.

**Fig. 2.**
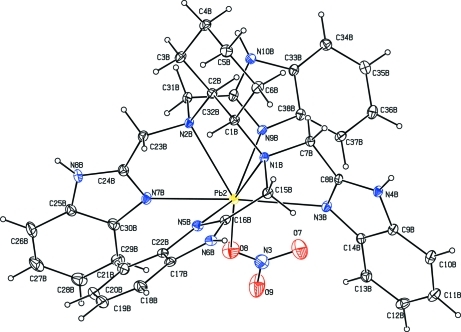
A view of the molecular structure of the Pb2 containing cation with displacement ellipsoids drawn at the 10% probability level.

**Fig. 3.**
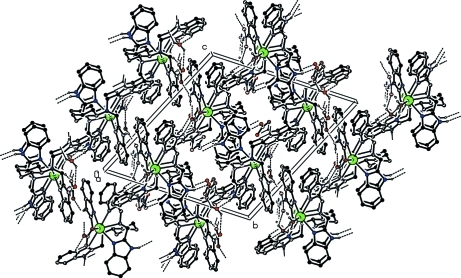
The crystal packing showing hydrogen bonds shown as dashed lines.

## supplementary crystallographic information

### Comment

Lead(II) complexes are interesting when considering the coordination and stereoactivity of heavy metals (Schwerdtheger *et al.*,1992). In recent years, lead(II) complexes with N-donor ligands have been widely studied (Bazzicalupi *et al.*, 1999; Kavallieratos *et al.*, 2005). In a continuation of our studies of lead(II) complexes containing poly-benzimidazole groups (Zhang *et al.*, 2007), the title compound, (I), has been prepared and its crystal structure is presented here.

In (I) (Fig.1), both Pb^II^ ions are coordinated in a distorted monocapped trigonal-prismatic environment by four benzimidazole N atoms and two amino N atoms of the ligand CTB. In one complex cation a half occupancy H_2_O ligand is present and in the other a nitrate ligand completes the coordination. The seven donor atoms occupy a space around the Pb^II^ ion and an additional space is occupied by the lone pair (Byriel *et al.*, 1992). In the crystal (Fig. 2) the components of the structure are linked by N—H···O and O—H···O hydrogen bonds to form a three-dimensional network.

### Experimental

All reagents and solvents were used as obtained without further purification. The ligand CTB was synthesized according to the literature methods (Hendriks *et al.*, 1982).Compound (I) was synthesized by reaction of CTB (0.64 g, 1 mmol) and Pb(NO_3_)_2_ (0.33 g, 1 mmol) in 95% ethanol (30 ml) at 333 K for 6 h. The solution was cooled to room temperature, filtered and evaporated to obtain the product (yield 80%). Crystals of (I) were grown from an ethanol solution by slow evaporation.Elemental analysis calculated: C 45.92, H 4.13, N 14.50%; found: C 45.76, H 4.49, N 14.84%.

### Refinement

All hydrogen atoms were placed in positions [*C*—*H*(methylene) = 0.97 Å, C—H(methyne) = 0.98 Å, *N*—*H*(amine)= 0.86Å and C—H(aromatic)= 0.93 Å] and included in the refinement in a riding-motion approximation, with *U*_iso_(*H*) = 1.2*U*_eq_(C). Hydrogen atoms bonded to oxygen atoms were placed calculated ideal positions (for H-bonds) with O—H = 0.84Å and *U*_iso_(*H*)=1.5*U*_eq_(O).

### Crystal data

**Table:**

[Pb(NO_3_)(C_38_H_38_N_10_)][Pb(C_38_H_38_N_10_)(H_2_O)_0.5_](NO_3_)_3_·2H_2_O	*Z* = 2
*M**_r_* = 1978.0	*F*(000) = 1972
Triclinic, *P*1	*D*_x_ = 1.653 Mg m^−^^3^
Hall symbol: -P 1	Mo *K*α radiation, λ = 0.71073 Å
*a* = 12.5485 (8) Å	Cell parameters from 6234 reflections
*b* = 18.5378 (11) Å	θ = 2.3–21.8°
*c* = 19.2839 (12) Å	µ = 4.31 mm^−^^1^
α = 64.516 (1)°	*T* = 298 K
β = 81.957 (2)°	Block, pale-yellow
γ = 79.675 (1)°	0.20 × 0.10 × 0.10 mm
*V* = 3973.9 (4) Å^3^	

### Data collection

**Table:**

Bruker SMART CCD diffractometer	15546 independent reflections
Radiation source: fine-focus sealed tube	11020 reflections with *I* > 2σ(*I*)
graphite	*R*_int_ = 0.054
φ and ω scans	θ_max_ = 26.0°, θ_min_ = 1.7°
Absorption correction: multi-scan (*SADABS*; Sheldrick, 1996)	*h* = −15→15
*T*_min_ = 0.479, *T*_max_ = 0.672	*k* = −22→22
41656 measured reflections	*l* = −23→23

### Refinement

**Table:**

Refinement on *F*^2^	Primary atom site location: structure-invariant direct methods
Least-squares matrix: full	Secondary atom site location: difference Fourier map
*R*[*F*^2^ > 2σ(*F*^2^)] = 0.045	Hydrogen site location: inferred from neighbouring sites
*wR*(*F*^2^) = 0.101	H-atom parameters constrained
*S* = 0.96	*w* = 1/[σ^2^(*F*_o_^2^) + (0.0424*P*)^2^] where *P* = (*F*_o_^2^ + 2*F*_c_^2^)/3
15546 reflections	(Δ/σ)_max_ = 0.004
1051 parameters	Δρ_max_ = 1.81 e Å^−^^3^
0 restraints	Δρ_min_ = −0.54 e Å^−^^3^

### Special details

**Table:**

Geometry. All esds (except the esd in the dihedral angle between two l.s. planes) are estimated using the full covariance matrix. The cell esds are taken into account individually in the estimation of esds in distances, angles and torsion angles; correlations between esds in cell parameters are only used when they are defined by crystal symmetry. An approximate (isotropic) treatment of cell esds is used for estimating esds involving l.s. planes.
Refinement. Refinement of F^2^ against ALL reflections. The weighted R-factor wR and goodness of fit S are based on F^2^, conventional R-factors R are based on F, with F set to zero for negative F^2^. The threshold expression of F^2^ > 2sigma(F^2^) is used only for calculating R-factors(gt) etc. and is not relevant to the choice of reflections for refinement. R-factors based on F^2^ are statistically about twice as large as those based on F, and R- factors based on ALL data will be even larger.

### Fractional atomic coordinates and isotropic or equivalent isotropic displacement parameters (Å^2^)

**Table:**

	*x*	*y*	*z*	*U*_iso_*/*U*_eq_	Occ. (<1)
Pb1	0.309639 (17)	0.243463 (14)	0.136457 (13)	0.03888 (8)	
C1A	0.0342 (4)	0.3300 (4)	0.1408 (3)	0.0370 (14)	
H1A	0.0118	0.2862	0.1889	0.044*	
C2A	0.0929 (4)	0.3804 (3)	0.1633 (3)	0.0361 (14)	
H2A	0.1120	0.4259	0.1155	0.043*	
C3A	0.0170 (5)	0.4164 (4)	0.2130 (3)	0.0456 (16)	
H3A1	−0.0046	0.3732	0.2609	0.055*	
H3A2	0.0556	0.4498	0.2253	0.055*	
C4A	−0.0831 (5)	0.4665 (4)	0.1727 (4)	0.0542 (18)	
H4A1	−0.1299	0.4877	0.2061	0.065*	
H4A2	−0.0623	0.5118	0.1262	0.065*	
C5A	−0.1429 (5)	0.4166 (4)	0.1526 (4)	0.0539 (18)	
H5A1	−0.2051	0.4500	0.1246	0.065*	
H5A2	−0.1694	0.3743	0.1994	0.065*	
C6A	−0.0698 (5)	0.3789 (4)	0.1033 (4)	0.0498 (17)	
H6A1	−0.1097	0.3440	0.0939	0.060*	
H6A2	−0.0510	0.4213	0.0539	0.060*	
C7A	0.1821 (4)	0.2867 (4)	0.2825 (3)	0.0413 (15)	
H7A1	0.1272	0.2523	0.2912	0.050*	
H7A2	0.1559	0.3213	0.3090	0.050*	
C8A	0.2844 (5)	0.2358 (4)	0.3148 (3)	0.0408 (15)	
C9A	0.4048 (6)	0.1643 (4)	0.3983 (4)	0.0524 (17)	
C10A	0.4669 (6)	0.1237 (4)	0.4619 (4)	0.068 (2)	
H10A	0.4426	0.1252	0.5091	0.081*	
C11A	0.5644 (7)	0.0817 (5)	0.4524 (5)	0.079 (2)	
H11A	0.6078	0.0546	0.4938	0.095*	
C12A	0.6001 (6)	0.0785 (5)	0.3827 (5)	0.075 (2)	
H12A	0.6669	0.0490	0.3785	0.091*	
C13A	0.5396 (6)	0.1179 (4)	0.3189 (5)	0.066 (2)	
H13A	0.5637	0.1147	0.2723	0.080*	
C14A	0.4406 (5)	0.1627 (4)	0.3277 (4)	0.0444 (15)	
C15A	0.2697 (5)	0.3977 (4)	0.1865 (3)	0.0393 (14)	
H15A	0.3316	0.3724	0.2177	0.047*	
H15B	0.2300	0.4391	0.2018	0.047*	
C16A	0.3081 (4)	0.4349 (4)	0.1035 (3)	0.0374 (14)	
C17A	0.3583 (4)	0.5257 (4)	−0.0092 (3)	0.0401 (15)	
C18A	0.3864 (5)	0.5943 (4)	−0.0725 (4)	0.0528 (18)	
H18A	0.3756	0.6446	−0.0711	0.063*	
C19A	0.4306 (5)	0.5836 (5)	−0.1369 (4)	0.0541 (18)	
H19A	0.4507	0.6280	−0.1803	0.065*	
C20A	0.4465 (5)	0.5096 (5)	−0.1398 (4)	0.059 (2)	
H20A	0.4766	0.5055	−0.1850	0.071*	
C21A	0.4190 (5)	0.4410 (4)	−0.0771 (4)	0.0538 (18)	
H21A	0.4303	0.3909	−0.0790	0.065*	
C22A	0.3730 (5)	0.4506 (4)	−0.0103 (3)	0.0441 (15)	
C23A	0.0635 (5)	0.2186 (3)	0.1013 (3)	0.0425 (15)	
H23A	0.0969	0.2027	0.0606	0.051*	
H23B	−0.0142	0.2306	0.0964	0.051*	
C24A	0.0871 (5)	0.1509 (3)	0.1782 (3)	0.0370 (14)	
C25A	0.0710 (5)	0.0383 (4)	0.2811 (3)	0.0427 (15)	
C26A	0.0384 (6)	−0.0314 (4)	0.3410 (4)	0.0555 (18)	
H26A	−0.0223	−0.0527	0.3390	0.067*	
C27A	0.1001 (7)	−0.0664 (4)	0.4022 (4)	0.067 (2)	
H27A	0.0812	−0.1132	0.4432	0.081*	
C28A	0.1896 (7)	−0.0355 (5)	0.4062 (4)	0.068 (2)	
H28A	0.2294	−0.0619	0.4495	0.082*	
C29A	0.2219 (6)	0.0339 (4)	0.3473 (4)	0.0588 (19)	
H29A	0.2827	0.0544	0.3502	0.071*	
C30A	0.1604 (5)	0.0716 (4)	0.2840 (3)	0.0406 (15)	
C31A	0.1151 (5)	0.3471 (4)	0.0103 (3)	0.0405 (14)	
H31A	0.1402	0.3959	0.0049	0.049*	
H31B	0.0441	0.3618	−0.0101	0.049*	
C32A	0.1927 (5)	0.3089 (4)	−0.0350 (3)	0.0399 (15)	
C33A	0.2502 (5)	0.2615 (4)	−0.1212 (3)	0.0436 (16)	
C34A	0.2645 (7)	0.2342 (4)	−0.1798 (4)	0.066 (2)	
H34A	0.2098	0.2442	−0.2120	0.079*	
C35A	0.3646 (8)	0.1916 (5)	−0.1867 (5)	0.077 (3)	
H35A	0.3769	0.1710	−0.2238	0.093*	
C36A	0.4473 (6)	0.1787 (5)	−0.1404 (5)	0.069 (2)	
H36A	0.5138	0.1502	−0.1475	0.083*	
C37A	0.4328 (5)	0.2070 (4)	−0.0844 (4)	0.0589 (19)	
H37A	0.4891	0.1993	−0.0542	0.071*	
C38A	0.3326 (5)	0.2470 (4)	−0.0739 (3)	0.0470 (16)	
N1A	0.1984 (3)	0.3365 (3)	0.1996 (2)	0.0329 (11)	
N2A	0.1062 (3)	0.2905 (3)	0.0936 (2)	0.0356 (11)	
N3A	0.3627 (4)	0.2091 (3)	0.2753 (3)	0.0496 (14)	
N4A	0.3048 (4)	0.2106 (3)	0.3888 (3)	0.0475 (13)	
H4A	0.2632	0.2213	0.4235	0.057*	
N5A	0.3399 (4)	0.3936 (3)	0.0622 (3)	0.0418 (12)	
N6A	0.3152 (4)	0.5140 (3)	0.0642 (3)	0.0399 (12)	
H6A	0.2965	0.5505	0.0814	0.048*	
N7A	0.1693 (4)	0.1431 (3)	0.2178 (3)	0.0416 (12)	
N8A	0.0262 (4)	0.0905 (3)	0.2125 (3)	0.0441 (13)	
H8A	−0.0301	0.0852	0.1950	0.053*	
N9A	0.2930 (4)	0.2771 (3)	−0.0182 (3)	0.0439 (13)	
N10A	0.1621 (4)	0.3014 (3)	−0.0957 (3)	0.0462 (13)	
H10D	0.0996	0.3183	−0.1146	0.055*	
Pb2	0.172648 (17)	0.747523 (13)	0.384700 (12)	0.03671 (8)	
C1B	0.4554 (4)	0.6667 (4)	0.3638 (3)	0.0379 (14)	
H1B	0.4740	0.7125	0.3161	0.045*	
C2B	0.3942 (4)	0.6170 (3)	0.3412 (3)	0.0364 (14)	
H2B	0.3797	0.5694	0.3884	0.044*	
C3B	0.4658 (5)	0.5861 (4)	0.2862 (3)	0.0463 (16)	
H3B1	0.4262	0.5533	0.2738	0.056*	
H3B2	0.4830	0.6315	0.2387	0.056*	
C4B	0.5709 (5)	0.5362 (4)	0.3211 (4)	0.0503 (17)	
H4B1	0.5543	0.4888	0.3668	0.060*	
H4B2	0.6149	0.5183	0.2844	0.060*	
C5B	0.6333 (5)	0.5852 (4)	0.3417 (4)	0.0533 (18)	
H5B1	0.6557	0.6299	0.2954	0.064*	
H5B2	0.6981	0.5520	0.3663	0.064*	
C6B	0.5636 (4)	0.6171 (4)	0.3958 (4)	0.0481 (16)	
H6B1	0.6041	0.6505	0.4066	0.058*	
H6B2	0.5483	0.5719	0.4441	0.058*	
C7B	0.3850 (5)	0.6437 (3)	0.4972 (3)	0.0375 (14)	
H7B1	0.4581	0.6277	0.5142	0.045*	
H7B2	0.3570	0.5959	0.5028	0.045*	
C8B	0.3151 (5)	0.6802 (4)	0.5464 (3)	0.0373 (14)	
C9B	0.2712 (5)	0.7295 (4)	0.6326 (3)	0.0413 (15)	
C10B	0.2678 (6)	0.7568 (4)	0.6903 (4)	0.0565 (18)	
H10B	0.3264	0.7449	0.7199	0.068*	
C11B	0.1724 (6)	0.8022 (4)	0.7006 (4)	0.063 (2)	
H11B	0.1669	0.8235	0.7369	0.076*	
C12B	0.0848 (6)	0.8169 (4)	0.6582 (4)	0.0585 (19)	
H12B	0.0212	0.8468	0.6677	0.070*	
C13B	0.0881 (5)	0.7889 (4)	0.6024 (4)	0.0510 (17)	
H13B	0.0279	0.7983	0.5750	0.061*	
C14B	0.1852 (5)	0.7458 (3)	0.5886 (3)	0.0387 (14)	
C15B	0.4302 (5)	0.7746 (3)	0.4066 (3)	0.0410 (15)	
H15C	0.5085	0.7639	0.4082	0.049*	
H15D	0.4001	0.7888	0.4489	0.049*	
C16B	0.4008 (5)	0.8430 (4)	0.3324 (3)	0.0381 (14)	
C17B	0.4123 (5)	0.9555 (4)	0.2278 (4)	0.0447 (16)	
C18B	0.4399 (6)	1.0254 (4)	0.1675 (4)	0.0586 (19)	
H18B	0.5014	1.0472	0.1671	0.070*	
C19B	0.3730 (7)	1.0612 (4)	0.1085 (4)	0.067 (2)	
H19B	0.3898	1.1080	0.0666	0.081*	
C20B	0.2816 (7)	1.0300 (4)	0.1094 (4)	0.066 (2)	
H20B	0.2377	1.0565	0.0682	0.079*	
C21B	0.2524 (5)	0.9603 (4)	0.1694 (4)	0.0521 (17)	
H21B	0.1905	0.9393	0.1693	0.063*	
C22B	0.3200 (5)	0.9227 (4)	0.2304 (3)	0.0410 (15)	
C23B	0.2950 (5)	0.7147 (4)	0.2284 (3)	0.0415 (15)	
H23C	0.3187	0.6831	0.1986	0.050*	
H23D	0.3497	0.7492	0.2197	0.050*	
C24B	0.1909 (5)	0.7653 (4)	0.2016 (3)	0.0436 (15)	
C25B	0.0691 (6)	0.8424 (4)	0.1206 (4)	0.0494 (17)	
C26B	0.0058 (7)	0.8873 (5)	0.0583 (4)	0.075 (2)	
H26B	0.0296	0.8910	0.0091	0.089*	
C27B	−0.0919 (7)	0.9254 (5)	0.0723 (5)	0.081 (3)	
H27B	−0.1352	0.9568	0.0315	0.097*	
C28B	−0.1297 (6)	0.9190 (5)	0.1465 (5)	0.075 (2)	
H28B	−0.1973	0.9460	0.1538	0.090*	
C29B	−0.0690 (5)	0.8738 (4)	0.2078 (4)	0.0607 (19)	
H29B	−0.0943	0.8693	0.2570	0.073*	
C30B	0.0327 (5)	0.8342 (4)	0.1948 (4)	0.0487 (16)	
C31B	0.2142 (5)	0.5989 (3)	0.3240 (3)	0.0378 (14)	
H31C	0.2528	0.5589	0.3064	0.045*	
H31D	0.1505	0.6246	0.2948	0.045*	
C32B	0.1804 (4)	0.5590 (4)	0.4072 (3)	0.0375 (14)	
C33B	0.1377 (4)	0.4662 (4)	0.5217 (3)	0.0387 (14)	
C34B	0.1160 (5)	0.3953 (4)	0.5852 (4)	0.0478 (16)	
H34B	0.1270	0.3454	0.5828	0.057*	
C35B	0.0772 (5)	0.4049 (4)	0.6514 (4)	0.0480 (16)	
H35B	0.0614	0.3596	0.6955	0.058*	
C36B	0.0608 (5)	0.4786 (4)	0.6554 (4)	0.0514 (17)	
H36B	0.0337	0.4816	0.7016	0.062*	
C37B	0.0834 (5)	0.5474 (4)	0.5928 (3)	0.0468 (16)	
H37B	0.0724	0.5970	0.5958	0.056*	
C38B	0.1235 (4)	0.5406 (4)	0.5244 (3)	0.0375 (14)	
N1B	0.3879 (4)	0.7014 (3)	0.4154 (2)	0.0348 (11)	
N2B	0.2853 (4)	0.6599 (3)	0.3109 (2)	0.0343 (11)	
N3B	0.2146 (4)	0.7141 (3)	0.5338 (3)	0.0400 (12)	
N4B	0.3540 (4)	0.6876 (3)	0.6046 (3)	0.0388 (12)	
H4B	0.4180	0.6697	0.6209	0.047*	
N5B	0.3156 (4)	0.8509 (3)	0.2963 (3)	0.0416 (12)	
N6B	0.4624 (4)	0.9034 (3)	0.2947 (3)	0.0423 (12)	
H6B	0.5211	0.9086	0.3094	0.051*	
N7B	0.1109 (4)	0.7846 (3)	0.2456 (3)	0.0436 (13)	
N8B	0.1702 (4)	0.7982 (3)	0.1266 (3)	0.0488 (14)	
H8B	0.2123	0.7924	0.0895	0.059*	
N9B	0.1515 (4)	0.5989 (3)	0.4516 (3)	0.0379 (12)	
N10B	0.1746 (4)	0.4802 (3)	0.4461 (3)	0.0388 (12)	
H10C	0.1907	0.4445	0.4277	0.047*	
N1	0.1213 (5)	0.6318 (4)	0.1254 (3)	0.0542 (15)	
O1	0.1118 (4)	0.6597 (4)	0.0550 (3)	0.101 (2)	
O2	0.0413 (4)	0.6359 (3)	0.1681 (3)	0.0799 (16)	
O3	0.2134 (4)	0.6034 (3)	0.1498 (3)	0.0723 (15)	
N2	0.3698 (5)	0.3788 (3)	0.3680 (3)	0.0503 (14)	
O4	0.4432 (4)	0.3665 (3)	0.3234 (3)	0.0667 (14)	
O5	0.2748 (4)	0.4021 (3)	0.3498 (3)	0.0573 (12)	
O6	0.3909 (5)	0.3666 (5)	0.4328 (3)	0.113 (2)	
N3	−0.1203 (6)	0.7786 (4)	0.4582 (5)	0.0734 (19)	
O7	−0.0864 (7)	0.7406 (5)	0.5206 (4)	0.156 (3)	
O8	−0.0624 (5)	0.7890 (4)	0.4006 (4)	0.094 (2)	
O9	−0.2157 (5)	0.8032 (5)	0.4581 (4)	0.138 (3)	
N4	0.7687 (6)	0.0921 (6)	0.1731 (5)	0.089 (2)	
O10	0.6772 (5)	0.0942 (5)	0.1534 (4)	0.139 (3)	
O11	0.8327 (5)	0.1368 (5)	0.1286 (4)	0.119 (3)	
O12	0.7956 (6)	0.0428 (4)	0.2372 (4)	0.107 (2)	
O1A	0.5342 (10)	0.2224 (8)	0.0922 (9)	0.143 (6)	0.50
H1O	0.5450	0.2458	0.0441	0.214*	0.5
H2O	0.5794	0.1814	0.1127	0.214*	0.5
O13	0.7056 (3)	0.2379 (3)	−0.0076 (2)	0.141 (3)	
H3O	0.7054	0.1879	0.0174	0.211*	
H4O	0.7365	0.2741	−0.0068	0.211*	
O14	0.6727 (3)	0.9059 (3)	0.3234 (2)	0.0883 (17)	
H5O	0.7052	0.8754	0.3632	0.132*	
H6O	0.7074	0.9452	0.2984	0.132*	

### Atomic displacement parameters (Å^2^)

**Table:**

	*U*^11^	*U*^22^	*U*^33^	*U*^12^	*U*^13^	*U*^23^
Pb1	0.03274 (13)	0.04629 (16)	0.04183 (14)	−0.00123 (11)	−0.00493 (10)	−0.02312 (12)
C1A	0.029 (3)	0.046 (4)	0.039 (3)	0.001 (3)	−0.003 (3)	−0.023 (3)
C2A	0.032 (3)	0.042 (4)	0.039 (3)	−0.001 (3)	−0.008 (3)	−0.022 (3)
C3A	0.042 (4)	0.055 (4)	0.049 (4)	0.001 (3)	−0.005 (3)	−0.032 (3)
C4A	0.048 (4)	0.059 (5)	0.059 (4)	0.008 (3)	−0.009 (3)	−0.031 (4)
C5A	0.037 (4)	0.062 (5)	0.064 (4)	0.008 (3)	−0.008 (3)	−0.031 (4)
C6A	0.041 (4)	0.061 (5)	0.052 (4)	0.004 (3)	−0.012 (3)	−0.029 (4)
C7A	0.036 (3)	0.055 (4)	0.036 (3)	−0.010 (3)	−0.002 (3)	−0.021 (3)
C8A	0.040 (3)	0.047 (4)	0.039 (3)	−0.007 (3)	−0.006 (3)	−0.020 (3)
C9A	0.057 (4)	0.044 (4)	0.050 (4)	−0.004 (3)	−0.018 (3)	−0.010 (3)
C10A	0.072 (5)	0.066 (5)	0.055 (5)	−0.004 (4)	−0.025 (4)	−0.011 (4)
C11A	0.072 (6)	0.073 (6)	0.081 (6)	−0.003 (5)	−0.039 (5)	−0.013 (5)
C12A	0.052 (5)	0.059 (5)	0.103 (7)	0.007 (4)	−0.033 (5)	−0.019 (5)
C13A	0.051 (4)	0.066 (5)	0.081 (5)	−0.003 (4)	−0.006 (4)	−0.031 (4)
C14A	0.038 (4)	0.045 (4)	0.053 (4)	−0.003 (3)	−0.011 (3)	−0.021 (3)
C15A	0.040 (3)	0.049 (4)	0.035 (3)	−0.007 (3)	−0.006 (3)	−0.022 (3)
C16A	0.031 (3)	0.047 (4)	0.043 (4)	−0.008 (3)	−0.006 (3)	−0.025 (3)
C17A	0.028 (3)	0.053 (4)	0.039 (4)	−0.008 (3)	−0.006 (3)	−0.016 (3)
C18A	0.040 (4)	0.057 (5)	0.050 (4)	−0.005 (3)	−0.011 (3)	−0.010 (4)
C19A	0.041 (4)	0.076 (5)	0.040 (4)	−0.015 (4)	−0.004 (3)	−0.017 (4)
C20A	0.044 (4)	0.089 (6)	0.052 (4)	−0.021 (4)	0.008 (3)	−0.036 (4)
C21A	0.045 (4)	0.070 (5)	0.054 (4)	−0.013 (4)	0.007 (3)	−0.034 (4)
C22A	0.035 (3)	0.055 (4)	0.043 (4)	−0.008 (3)	−0.006 (3)	−0.019 (3)
C23A	0.046 (4)	0.044 (4)	0.041 (4)	−0.011 (3)	−0.005 (3)	−0.019 (3)
C24A	0.038 (3)	0.036 (4)	0.042 (4)	−0.004 (3)	−0.003 (3)	−0.021 (3)
C25A	0.047 (4)	0.037 (4)	0.043 (4)	−0.002 (3)	−0.002 (3)	−0.018 (3)
C26A	0.062 (5)	0.038 (4)	0.063 (5)	−0.010 (3)	−0.005 (4)	−0.016 (4)
C27A	0.089 (6)	0.043 (4)	0.064 (5)	−0.007 (4)	−0.010 (5)	−0.016 (4)
C28A	0.082 (6)	0.057 (5)	0.057 (5)	0.007 (4)	−0.027 (4)	−0.016 (4)
C29A	0.063 (5)	0.050 (5)	0.062 (5)	0.007 (4)	−0.022 (4)	−0.022 (4)
C30A	0.037 (3)	0.041 (4)	0.043 (4)	0.003 (3)	−0.002 (3)	−0.020 (3)
C31A	0.043 (4)	0.043 (4)	0.032 (3)	−0.004 (3)	−0.004 (3)	−0.014 (3)
C32A	0.051 (4)	0.040 (4)	0.028 (3)	−0.007 (3)	−0.003 (3)	−0.012 (3)
C33A	0.056 (4)	0.040 (4)	0.031 (3)	−0.012 (3)	0.009 (3)	−0.012 (3)
C34A	0.086 (6)	0.071 (5)	0.053 (4)	−0.020 (5)	0.011 (4)	−0.038 (4)
C35A	0.104 (7)	0.067 (6)	0.071 (6)	−0.027 (5)	0.036 (5)	−0.045 (5)
C36A	0.064 (5)	0.063 (5)	0.080 (6)	−0.010 (4)	0.026 (5)	−0.038 (5)
C37A	0.051 (4)	0.056 (5)	0.064 (5)	−0.005 (4)	0.009 (4)	−0.024 (4)
C38A	0.053 (4)	0.047 (4)	0.038 (4)	−0.006 (3)	0.006 (3)	−0.018 (3)
N1A	0.031 (2)	0.038 (3)	0.030 (3)	−0.003 (2)	−0.006 (2)	−0.014 (2)
N2A	0.033 (3)	0.046 (3)	0.032 (3)	−0.002 (2)	−0.005 (2)	−0.021 (2)
N3A	0.042 (3)	0.059 (4)	0.052 (3)	0.008 (3)	−0.015 (3)	−0.029 (3)
N4A	0.048 (3)	0.060 (4)	0.032 (3)	−0.004 (3)	−0.007 (2)	−0.016 (3)
N5A	0.041 (3)	0.049 (3)	0.044 (3)	−0.013 (2)	0.002 (2)	−0.026 (3)
N6A	0.036 (3)	0.045 (3)	0.044 (3)	−0.006 (2)	−0.005 (2)	−0.024 (3)
N7A	0.037 (3)	0.044 (3)	0.043 (3)	−0.003 (2)	−0.005 (2)	−0.017 (3)
N8A	0.041 (3)	0.046 (3)	0.052 (3)	−0.008 (3)	−0.003 (3)	−0.026 (3)
N9A	0.043 (3)	0.050 (3)	0.038 (3)	−0.002 (3)	0.001 (2)	−0.020 (3)
N10A	0.049 (3)	0.055 (4)	0.038 (3)	−0.003 (3)	−0.006 (2)	−0.022 (3)
Pb2	0.03524 (13)	0.04008 (15)	0.03724 (14)	−0.00111 (11)	−0.00663 (10)	−0.01866 (11)
C1B	0.033 (3)	0.044 (4)	0.042 (3)	−0.002 (3)	−0.002 (3)	−0.024 (3)
C2B	0.031 (3)	0.041 (4)	0.037 (3)	0.002 (3)	−0.007 (3)	−0.017 (3)
C3B	0.040 (4)	0.059 (4)	0.050 (4)	0.001 (3)	−0.004 (3)	−0.035 (3)
C4B	0.048 (4)	0.056 (4)	0.050 (4)	0.008 (3)	−0.006 (3)	−0.030 (3)
C5B	0.031 (3)	0.068 (5)	0.057 (4)	0.008 (3)	−0.004 (3)	−0.028 (4)
C6B	0.032 (3)	0.064 (5)	0.058 (4)	0.000 (3)	−0.009 (3)	−0.035 (4)
C7B	0.042 (3)	0.038 (4)	0.034 (3)	−0.001 (3)	−0.010 (3)	−0.016 (3)
C8B	0.043 (4)	0.044 (4)	0.025 (3)	−0.002 (3)	−0.003 (3)	−0.016 (3)
C9B	0.045 (4)	0.047 (4)	0.037 (3)	−0.010 (3)	0.002 (3)	−0.022 (3)
C10B	0.066 (5)	0.068 (5)	0.044 (4)	−0.017 (4)	−0.001 (4)	−0.030 (4)
C11B	0.075 (5)	0.071 (5)	0.061 (5)	−0.018 (4)	0.008 (4)	−0.045 (4)
C12B	0.053 (4)	0.058 (5)	0.069 (5)	−0.007 (4)	0.014 (4)	−0.036 (4)
C13B	0.043 (4)	0.060 (5)	0.045 (4)	−0.001 (3)	−0.005 (3)	−0.020 (3)
C14B	0.046 (4)	0.037 (4)	0.030 (3)	−0.007 (3)	0.004 (3)	−0.011 (3)
C15B	0.035 (3)	0.047 (4)	0.046 (4)	−0.004 (3)	−0.009 (3)	−0.022 (3)
C16B	0.041 (3)	0.038 (4)	0.040 (3)	−0.005 (3)	−0.001 (3)	−0.022 (3)
C17B	0.047 (4)	0.043 (4)	0.047 (4)	0.001 (3)	0.005 (3)	−0.026 (3)
C18B	0.065 (5)	0.043 (4)	0.061 (5)	−0.013 (4)	0.001 (4)	−0.015 (4)
C19B	0.095 (6)	0.042 (4)	0.050 (5)	−0.009 (4)	0.003 (4)	−0.007 (4)
C20B	0.087 (6)	0.049 (5)	0.050 (5)	0.008 (4)	−0.021 (4)	−0.012 (4)
C21B	0.057 (4)	0.048 (4)	0.052 (4)	−0.004 (3)	−0.008 (3)	−0.021 (4)
C22B	0.042 (4)	0.036 (4)	0.044 (4)	0.005 (3)	−0.005 (3)	−0.019 (3)
C23B	0.044 (4)	0.044 (4)	0.037 (3)	−0.005 (3)	−0.001 (3)	−0.019 (3)
C24B	0.047 (4)	0.044 (4)	0.041 (4)	−0.011 (3)	−0.007 (3)	−0.016 (3)
C25B	0.065 (5)	0.038 (4)	0.043 (4)	−0.012 (3)	−0.014 (3)	−0.010 (3)
C26B	0.088 (6)	0.073 (6)	0.048 (4)	−0.002 (5)	−0.030 (4)	−0.007 (4)
C27B	0.077 (6)	0.066 (6)	0.087 (7)	0.005 (5)	−0.050 (5)	−0.012 (5)
C28B	0.065 (5)	0.062 (5)	0.102 (7)	0.003 (4)	−0.037 (5)	−0.034 (5)
C29B	0.055 (4)	0.061 (5)	0.074 (5)	0.011 (4)	−0.027 (4)	−0.036 (4)
C30B	0.044 (4)	0.046 (4)	0.055 (4)	0.000 (3)	−0.022 (3)	−0.017 (3)
C31B	0.038 (3)	0.046 (4)	0.033 (3)	−0.007 (3)	−0.005 (3)	−0.019 (3)
C32B	0.029 (3)	0.054 (4)	0.036 (3)	−0.009 (3)	−0.003 (3)	−0.023 (3)
C33B	0.034 (3)	0.051 (4)	0.037 (3)	−0.009 (3)	−0.003 (3)	−0.022 (3)
C34B	0.050 (4)	0.044 (4)	0.050 (4)	−0.010 (3)	−0.006 (3)	−0.019 (3)
C35B	0.041 (4)	0.054 (4)	0.042 (4)	−0.013 (3)	−0.005 (3)	−0.011 (3)
C36B	0.046 (4)	0.071 (5)	0.039 (4)	−0.013 (4)	0.005 (3)	−0.025 (4)
C37B	0.051 (4)	0.047 (4)	0.048 (4)	−0.002 (3)	0.000 (3)	−0.028 (3)
C38B	0.033 (3)	0.048 (4)	0.034 (3)	−0.007 (3)	−0.003 (3)	−0.019 (3)
N1B	0.037 (3)	0.037 (3)	0.033 (3)	−0.002 (2)	−0.009 (2)	−0.017 (2)
N2B	0.033 (3)	0.044 (3)	0.032 (3)	−0.004 (2)	−0.006 (2)	−0.020 (2)
N3B	0.039 (3)	0.048 (3)	0.038 (3)	−0.005 (2)	−0.004 (2)	−0.023 (3)
N4B	0.036 (3)	0.050 (3)	0.036 (3)	−0.002 (2)	−0.009 (2)	−0.022 (2)
N5B	0.041 (3)	0.039 (3)	0.048 (3)	−0.007 (2)	−0.006 (2)	−0.020 (3)
N6B	0.034 (3)	0.041 (3)	0.056 (3)	−0.006 (2)	−0.004 (3)	−0.024 (3)
N7B	0.042 (3)	0.055 (4)	0.039 (3)	−0.004 (3)	−0.011 (2)	−0.023 (3)
N8B	0.055 (4)	0.053 (4)	0.030 (3)	−0.006 (3)	−0.009 (3)	−0.009 (3)
N9B	0.042 (3)	0.042 (3)	0.036 (3)	−0.003 (2)	−0.003 (2)	−0.022 (2)
N10B	0.043 (3)	0.038 (3)	0.041 (3)	−0.009 (2)	−0.005 (2)	−0.020 (2)
N1	0.061 (4)	0.065 (4)	0.049 (4)	−0.004 (3)	−0.010 (3)	−0.034 (3)
O1	0.077 (4)	0.175 (7)	0.055 (3)	−0.004 (4)	−0.016 (3)	−0.053 (4)
O2	0.065 (3)	0.106 (5)	0.062 (3)	−0.005 (3)	0.004 (3)	−0.033 (3)
O3	0.064 (3)	0.087 (4)	0.084 (4)	0.017 (3)	−0.031 (3)	−0.055 (3)
N2	0.049 (4)	0.057 (4)	0.055 (4)	0.001 (3)	−0.014 (3)	−0.032 (3)
O4	0.050 (3)	0.098 (4)	0.057 (3)	−0.002 (3)	−0.001 (2)	−0.041 (3)
O5	0.049 (3)	0.071 (3)	0.068 (3)	0.010 (2)	−0.022 (2)	−0.046 (3)
O6	0.073 (4)	0.211 (8)	0.085 (4)	0.017 (4)	−0.032 (3)	−0.097 (5)
N3	0.073 (5)	0.071 (5)	0.080 (5)	−0.013 (4)	−0.017 (5)	−0.030 (4)
O7	0.202 (9)	0.145 (7)	0.112 (6)	0.024 (6)	−0.079 (6)	−0.044 (5)
O8	0.070 (4)	0.108 (5)	0.097 (5)	−0.026 (3)	0.031 (4)	−0.041 (4)
O9	0.071 (4)	0.239 (9)	0.093 (5)	0.015 (5)	0.002 (4)	−0.074 (6)
N4	0.058 (5)	0.136 (8)	0.108 (7)	−0.019 (5)	−0.004 (5)	−0.083 (6)
O10	0.063 (4)	0.219 (9)	0.176 (7)	−0.033 (5)	−0.022 (5)	−0.109 (7)
O11	0.080 (5)	0.163 (7)	0.114 (5)	−0.053 (5)	−0.006 (4)	−0.044 (5)
O12	0.110 (5)	0.119 (6)	0.110 (5)	−0.030 (5)	−0.015 (4)	−0.058 (5)
O1A	0.088 (10)	0.103 (11)	0.192 (15)	−0.012 (8)	−0.007 (10)	−0.020 (10)
O13	0.180 (7)	0.134 (6)	0.092 (5)	−0.020 (5)	0.043 (5)	−0.048 (5)
O14	0.062 (3)	0.102 (5)	0.106 (4)	−0.010 (3)	−0.020 (3)	−0.044 (4)

### Geometric parameters (Å, °)

**Table:**

Pb1—N5A	2.596 (5)	C1B—H1B	0.9800
Pb1—N1A	2.623 (4)	C2B—N2B	1.508 (6)
Pb1—N3A	2.627 (5)	C2B—C3B	1.528 (7)
Pb1—N7A	2.629 (5)	C2B—H2B	0.9800
Pb1—N2A	2.671 (4)	C3B—C4B	1.524 (8)
Pb1—O1A	2.836 (13)	C3B—H3B1	0.9700
C1A—N2A	1.513 (7)	C3B—H3B2	0.9700
C1A—C2A	1.512 (7)	C4B—C5B	1.500 (8)
C1A—C6A	1.524 (7)	C4B—H4B1	0.9700
C1A—H1A	0.9800	C4B—H4B2	0.9700
C2A—N1A	1.512 (6)	C5B—C6B	1.514 (8)
C2A—C3A	1.530 (7)	C5B—H5B1	0.9700
C2A—H2A	0.9800	C5B—H5B2	0.9700
C3A—C4A	1.509 (8)	C6B—H6B1	0.9700
C3A—H3A1	0.9700	C6B—H6B2	0.9700
C3A—H3A2	0.9700	C7B—N1B	1.477 (7)
C4A—C5A	1.487 (9)	C7B—C8B	1.494 (7)
C4A—H4A1	0.9700	C7B—H7B1	0.9700
C4A—H4A2	0.9700	C7B—H7B2	0.9700
C5A—C6A	1.527 (8)	C8B—N3B	1.310 (7)
C5A—H5A1	0.9700	C8B—N4B	1.352 (6)
C5A—H5A2	0.9700	C9B—N4B	1.375 (7)
C6A—H6A1	0.9700	C9B—C14B	1.376 (8)
C6A—H6A2	0.9700	C9B—C10B	1.399 (8)
C7A—N1A	1.468 (7)	C10B—C11B	1.379 (9)
C7A—C8A	1.486 (8)	C10B—H10B	0.9300
C7A—H7A1	0.9700	C11B—C12B	1.380 (9)
C7A—H7A2	0.9700	C11B—H11B	0.9300
C8A—N3A	1.316 (7)	C12B—C13B	1.375 (9)
C8A—N4A	1.343 (7)	C12B—H12B	0.9300
C9A—N4A	1.374 (8)	C13B—C14B	1.392 (8)
C9A—C14A	1.385 (8)	C13B—H13B	0.9300
C9A—C10A	1.392 (9)	C14B—N3B	1.394 (7)
C10A—C11A	1.362 (10)	C15B—N1B	1.478 (7)
C10A—H10A	0.9300	C15B—C16B	1.487 (8)
C11A—C12A	1.378 (11)	C15B—H15C	0.9700
C11A—H11A	0.9300	C15B—H15D	0.9700
C12A—C13A	1.383 (10)	C16B—N5B	1.308 (7)
C12A—H12A	0.9300	C16B—N6B	1.354 (7)
C13A—C14A	1.398 (8)	C17B—C18B	1.381 (8)
C13A—H13A	0.9300	C17B—C22B	1.387 (8)
C14A—N3A	1.394 (7)	C17B—N6B	1.389 (7)
C15A—N1A	1.485 (7)	C18B—C19B	1.364 (10)
C15A—C16A	1.491 (8)	C18B—H18B	0.9300
C15A—H15A	0.9700	C19B—C20B	1.370 (10)
C15A—H15B	0.9700	C19B—H19B	0.9300
C16A—N5A	1.306 (7)	C20B—C21B	1.381 (9)
C16A—N6A	1.344 (7)	C20B—H20B	0.9300
C17A—N6A	1.380 (7)	C21B—C22B	1.395 (8)
C17A—C22A	1.381 (8)	C21B—H21B	0.9300
C17A—C18A	1.392 (8)	C22B—N5B	1.394 (7)
C18A—C19A	1.366 (9)	C23B—N2B	1.476 (7)
C18A—H18A	0.9300	C23B—C24B	1.478 (8)
C19A—C20A	1.375 (9)	C23B—H23C	0.9700
C19A—H19A	0.9300	C23B—H23D	0.9700
C20A—C21A	1.385 (9)	C24B—N7B	1.323 (7)
C20A—H20A	0.9300	C24B—N8B	1.350 (7)
C21A—C22A	1.405 (8)	C25B—N8B	1.371 (8)
C21A—H21A	0.9300	C25B—C30B	1.391 (9)
C22A—N5A	1.402 (7)	C25B—C26B	1.391 (9)
C23A—N2A	1.465 (7)	C26B—C27B	1.356 (11)
C23A—C24A	1.500 (8)	C26B—H26B	0.9300
C23A—H23A	0.9700	C27B—C28B	1.403 (11)
C23A—H23B	0.9700	C27B—H27B	0.9300
C24A—N7A	1.318 (7)	C28B—C29B	1.361 (9)
C24A—N8A	1.343 (7)	C28B—H28B	0.9300
C25A—N8A	1.385 (7)	C29B—C30B	1.400 (9)
C25A—C30A	1.392 (8)	C29B—H29B	0.9300
C25A—C26A	1.395 (8)	C30B—N7B	1.393 (7)
C26A—C27A	1.352 (9)	C31B—N2B	1.482 (7)
C26A—H26A	0.9300	C31B—C32B	1.484 (7)
C27A—C28A	1.374 (10)	C31B—H31C	0.9700
C27A—H27A	0.9300	C31B—H31D	0.9700
C28A—C29A	1.382 (10)	C32B—N9B	1.333 (7)
C28A—H28A	0.9300	C32B—N10B	1.334 (7)
C29A—C30A	1.383 (8)	C33B—C38B	1.382 (8)
C29A—H29A	0.9300	C33B—N10B	1.391 (7)
C30A—N7A	1.399 (7)	C33B—C34B	1.397 (8)
C31A—N2A	1.495 (7)	C34B—C35B	1.375 (8)
C31A—C32A	1.502 (8)	C34B—H34B	0.9300
C31A—H31A	0.9700	C35B—C36B	1.378 (9)
C31A—H31B	0.9700	C35B—H35B	0.9300
C32A—N9A	1.312 (7)	C36B—C37B	1.369 (8)
C32A—N10A	1.350 (7)	C36B—H36B	0.9300
C33A—N10A	1.371 (7)	C37B—C38B	1.394 (8)
C33A—C38A	1.389 (8)	C37B—H37B	0.9300
C33A—C34A	1.401 (8)	C38B—N9B	1.398 (7)
C34A—C35A	1.381 (10)	N4B—H4B	0.8600
C34A—H34A	0.9300	N6B—H6B	0.8600
C35A—C36A	1.385 (11)	N8B—H8B	0.8600
C35A—H35A	0.9300	N10B—H10C	0.8600
C36A—C37A	1.369 (10)	N1—O2	1.219 (7)
C36A—H36A	0.9300	N1—O1	1.242 (7)
C37A—C38A	1.378 (8)	N1—O3	1.247 (6)
C37A—H37A	0.9300	N2—O4	1.229 (6)
C38A—N9A	1.405 (7)	N2—O6	1.230 (7)
N4A—H4A	0.8600	N2—O5	1.238 (6)
N6A—H6A	0.8600	N3—O7	1.196 (8)
N8A—H8A	0.8600	N3—O8	1.200 (8)
N10A—H10D	0.8600	N3—O9	1.203 (8)
Pb2—N9B	2.536 (5)	N4—O11	1.226 (9)
Pb2—N7B	2.659 (5)	N4—O12	1.228 (9)
Pb2—N2B	2.695 (4)	N4—O10	1.248 (8)
Pb2—N5B	2.713 (5)	O1A—H1O	0.8400
Pb2—N1B	2.750 (4)	O1A—H2O	0.8401
Pb2—N3B	2.776 (4)	O13—H3O	0.8401
Pb2—O8	2.921 (6)	O13—H4O	0.8399
C1B—N1B	1.497 (7)	O14—H5O	0.8400
C1B—C2B	1.523 (7)	O14—H6O	0.8399
C1B—C6B	1.539 (7)		
			
N5A—Pb1—N1A	66.76 (14)	N2B—Pb2—N3B	120.70 (14)
N5A—Pb1—N3A	102.37 (16)	N5B—Pb2—N3B	103.35 (14)
N1A—Pb1—N3A	65.63 (14)	N1B—Pb2—N3B	65.33 (13)
N5A—Pb1—N7A	145.68 (15)	N9B—Pb2—O8	90.00 (16)
N1A—Pb1—N7A	84.46 (14)	N7B—Pb2—O8	76.64 (17)
N3A—Pb1—N7A	80.78 (16)	N2B—Pb2—O8	127.97 (16)
N5A—Pb1—N2A	86.97 (15)	N5B—Pb2—O8	125.86 (16)
N1A—Pb1—N2A	68.01 (13)	N1B—Pb2—O8	162.66 (16)
N3A—Pb1—N2A	123.54 (14)	N3B—Pb2—O8	97.35 (16)
N7A—Pb1—N2A	64.27 (14)	N1B—C1B—C2B	113.1 (4)
N5A—Pb1—O1A	80.8 (3)	N1B—C1B—C6B	114.0 (5)
N1A—Pb1—O1A	130.9 (3)	C2B—C1B—C6B	109.6 (5)
N3A—Pb1—O1A	88.2 (3)	N1B—C1B—H1B	106.5
N7A—Pb1—O1A	133.5 (3)	C2B—C1B—H1B	106.5
N2A—Pb1—O1A	147.9 (3)	C6B—C1B—H1B	106.5
N2A—C1A—C2A	113.2 (4)	N2B—C2B—C1B	113.5 (5)
N2A—C1A—C6A	113.5 (4)	N2B—C2B—C3B	112.6 (4)
C2A—C1A—C6A	110.4 (5)	C1B—C2B—C3B	110.6 (5)
N2A—C1A—H1A	106.4	N2B—C2B—H2B	106.5
C2A—C1A—H1A	106.4	C1B—C2B—H2B	106.5
C6A—C1A—H1A	106.4	C3B—C2B—H2B	106.5
N1A—C2A—C1A	113.6 (5)	C4B—C3B—C2B	111.7 (5)
N1A—C2A—C3A	112.5 (4)	C4B—C3B—H3B1	109.3
C1A—C2A—C3A	110.7 (5)	C2B—C3B—H3B1	109.3
N1A—C2A—H2A	106.5	C4B—C3B—H3B2	109.3
C1A—C2A—H2A	106.5	C2B—C3B—H3B2	109.3
C3A—C2A—H2A	106.5	H3B1—C3B—H3B2	107.9
C4A—C3A—C2A	111.8 (5)	C5B—C4B—C3B	110.4 (5)
C4A—C3A—H3A1	109.2	C5B—C4B—H4B1	109.6
C2A—C3A—H3A1	109.2	C3B—C4B—H4B1	109.6
C4A—C3A—H3A2	109.2	C5B—C4B—H4B2	109.6
C2A—C3A—H3A2	109.2	C3B—C4B—H4B2	109.6
H3A1—C3A—H3A2	107.9	H4B1—C4B—H4B2	108.1
C5A—C4A—C3A	110.1 (5)	C4B—C5B—C6B	110.1 (5)
C5A—C4A—H4A1	109.6	C4B—C5B—H5B1	109.6
C3A—C4A—H4A1	109.6	C6B—C5B—H5B1	109.6
C5A—C4A—H4A2	109.6	C4B—C5B—H5B2	109.6
C3A—C4A—H4A2	109.6	C6B—C5B—H5B2	109.6
H4A1—C4A—H4A2	108.2	H5B1—C5B—H5B2	108.1
C4A—C5A—C6A	111.0 (5)	C5B—C6B—C1B	113.6 (5)
C4A—C5A—H5A1	109.4	C5B—C6B—H6B1	108.8
C6A—C5A—H5A1	109.4	C1B—C6B—H6B1	108.8
C4A—C5A—H5A2	109.4	C5B—C6B—H6B2	108.8
C6A—C5A—H5A2	109.4	C1B—C6B—H6B2	108.8
H5A1—C5A—H5A2	108.0	H6B1—C6B—H6B2	107.7
C1A—C6A—C5A	112.8 (5)	N1B—C7B—C8B	111.1 (5)
C1A—C6A—H6A1	109.0	N1B—C7B—H7B1	109.4
C5A—C6A—H6A1	109.0	C8B—C7B—H7B1	109.4
C1A—C6A—H6A2	109.0	N1B—C7B—H7B2	109.4
C5A—C6A—H6A2	109.0	C8B—C7B—H7B2	109.4
H6A1—C6A—H6A2	107.8	H7B1—C7B—H7B2	108.0
N1A—C7A—C8A	111.5 (5)	N3B—C8B—N4B	113.4 (5)
N1A—C7A—H7A1	109.3	N3B—C8B—C7B	124.2 (5)
C8A—C7A—H7A1	109.3	N4B—C8B—C7B	122.2 (5)
N1A—C7A—H7A2	109.3	N4B—C9B—C14B	105.9 (5)
C8A—C7A—H7A2	109.3	N4B—C9B—C10B	131.0 (6)
H7A1—C7A—H7A2	108.0	C14B—C9B—C10B	123.0 (6)
N3A—C8A—N4A	113.0 (5)	C11B—C10B—C9B	115.9 (6)
N3A—C8A—C7A	124.2 (5)	C11B—C10B—H10B	122.1
N4A—C8A—C7A	122.8 (5)	C9B—C10B—H10B	122.1
N4A—C9A—C14A	106.3 (5)	C10B—C11B—C12B	121.4 (6)
N4A—C9A—C10A	132.2 (7)	C10B—C11B—H11B	119.3
C14A—C9A—C10A	121.5 (7)	C12B—C11B—H11B	119.3
C11A—C10A—C9A	117.7 (7)	C13B—C12B—C11B	122.4 (6)
C11A—C10A—H10A	121.2	C13B—C12B—H12B	118.8
C9A—C10A—H10A	121.2	C11B—C12B—H12B	118.8
C10A—C11A—C12A	121.3 (7)	C12B—C13B—C14B	117.2 (6)
C10A—C11A—H11A	119.3	C12B—C13B—H13B	121.4
C12A—C11A—H11A	119.3	C14B—C13B—H13B	121.4
C11A—C12A—C13A	122.1 (7)	C9B—C14B—C13B	120.0 (6)
C11A—C12A—H12A	118.9	C9B—C14B—N3B	109.7 (5)
C13A—C12A—H12A	118.9	C13B—C14B—N3B	130.2 (6)
C12A—C13A—C14A	116.8 (7)	N1B—C15B—C16B	110.6 (5)
C12A—C13A—H13A	121.6	N1B—C15B—H15C	109.5
C14A—C13A—H13A	121.6	C16B—C15B—H15C	109.5
C9A—C14A—N3A	108.6 (5)	N1B—C15B—H15D	109.5
C9A—C14A—C13A	120.5 (6)	C16B—C15B—H15D	109.5
N3A—C14A—C13A	130.9 (6)	H15C—C15B—H15D	108.1
N1A—C15A—C16A	109.8 (4)	N5B—C16B—N6B	113.2 (5)
N1A—C15A—H15A	109.7	N5B—C16B—C15B	124.6 (5)
C16A—C15A—H15A	109.7	N6B—C16B—C15B	122.2 (5)
N1A—C15A—H15B	109.7	C18B—C17B—C22B	122.6 (6)
C16A—C15A—H15B	109.7	C18B—C17B—N6B	132.1 (6)
H15A—C15A—H15B	108.2	C22B—C17B—N6B	105.3 (5)
N5A—C16A—N6A	113.3 (5)	C19B—C18B—C17B	117.0 (7)
N5A—C16A—C15A	123.4 (6)	C19B—C18B—H18B	121.5
N6A—C16A—C15A	123.3 (5)	C17B—C18B—H18B	121.5
N6A—C17A—C22A	105.5 (5)	C18B—C19B—C20B	121.6 (7)
N6A—C17A—C18A	131.8 (6)	C18B—C19B—H19B	119.2
C22A—C17A—C18A	122.7 (6)	C20B—C19B—H19B	119.2
C19A—C18A—C17A	116.3 (7)	C19B—C20B—C21B	122.1 (7)
C19A—C18A—H18A	121.9	C19B—C20B—H20B	118.9
C17A—C18A—H18A	121.9	C21B—C20B—H20B	118.9
C18A—C19A—C20A	122.4 (7)	C20B—C21B—C22B	117.1 (7)
C18A—C19A—H19A	118.8	C20B—C21B—H21B	121.5
C20A—C19A—H19A	118.8	C22B—C21B—H21B	121.5
C19A—C20A—C21A	121.8 (6)	C17B—C22B—N5B	109.6 (5)
C19A—C20A—H20A	119.1	C17B—C22B—C21B	119.6 (6)
C21A—C20A—H20A	119.1	N5B—C22B—C21B	130.7 (6)
C20A—C21A—C22A	116.8 (7)	N2B—C23B—C24B	111.9 (5)
C20A—C21A—H21A	121.6	N2B—C23B—H23C	109.2
C22A—C21A—H21A	121.6	C24B—C23B—H23C	109.2
C17A—C22A—N5A	109.4 (5)	N2B—C23B—H23D	109.2
C17A—C22A—C21A	120.0 (6)	C24B—C23B—H23D	109.2
N5A—C22A—C21A	130.6 (6)	H23C—C23B—H23D	107.9
N2A—C23A—C24A	109.9 (5)	N7B—C24B—N8B	113.2 (6)
N2A—C23A—H23A	109.7	N7B—C24B—C23B	125.5 (5)
C24A—C23A—H23A	109.7	N8B—C24B—C23B	121.3 (6)
N2A—C23A—H23B	109.7	N8B—C25B—C30B	105.9 (5)
C24A—C23A—H23B	109.7	N8B—C25B—C26B	132.3 (7)
H23A—C23A—H23B	108.2	C30B—C25B—C26B	121.8 (7)
N7A—C24A—N8A	113.5 (5)	C27B—C26B—C25B	117.2 (7)
N7A—C24A—C23A	124.5 (5)	C27B—C26B—H26B	121.4
N8A—C24A—C23A	122.0 (5)	C25B—C26B—H26B	121.4
N8A—C25A—C30A	105.5 (5)	C26B—C27B—C28B	122.0 (7)
N8A—C25A—C26A	131.9 (6)	C26B—C27B—H27B	119.0
C30A—C25A—C26A	122.6 (6)	C28B—C27B—H27B	119.0
C27A—C26A—C25A	116.1 (7)	C29B—C28B—C27B	121.0 (8)
C27A—C26A—H26A	121.9	C29B—C28B—H28B	119.5
C25A—C26A—H26A	121.9	C27B—C28B—H28B	119.5
C26A—C27A—C28A	122.6 (7)	C28B—C29B—C30B	118.2 (7)
C26A—C27A—H27A	118.7	C28B—C29B—H29B	120.9
C28A—C27A—H27A	118.7	C30B—C29B—H29B	120.9
C27A—C28A—C29A	121.6 (7)	C25B—C30B—N7B	109.5 (6)
C27A—C28A—H28A	119.2	C25B—C30B—C29B	119.8 (6)
C29A—C28A—H28A	119.2	N7B—C30B—C29B	130.7 (6)
C28A—C29A—C30A	117.5 (7)	N2B—C31B—C32B	109.9 (4)
C28A—C29A—H29A	121.3	N2B—C31B—H31C	109.7
C30A—C29A—H29A	121.3	C32B—C31B—H31C	109.7
C29A—C30A—C25A	119.6 (6)	N2B—C31B—H31D	109.7
C29A—C30A—N7A	131.0 (6)	C32B—C31B—H31D	109.7
C25A—C30A—N7A	109.4 (5)	H31C—C31B—H31D	108.2
N2A—C31A—C32A	110.9 (5)	N9B—C32B—N10B	112.2 (5)
N2A—C31A—H31A	109.5	N9B—C32B—C31B	123.1 (5)
C32A—C31A—H31A	109.5	N10B—C32B—C31B	124.7 (5)
N2A—C31A—H31B	109.5	C38B—C33B—N10B	105.5 (5)
C32A—C31A—H31B	109.5	C38B—C33B—C34B	123.4 (5)
H31A—C31A—H31B	108.1	N10B—C33B—C34B	131.1 (6)
N9A—C32A—N10A	113.9 (5)	C35B—C34B—C33B	114.8 (6)
N9A—C32A—C31A	124.3 (5)	C35B—C34B—H34B	122.6
N10A—C32A—C31A	121.8 (5)	C33B—C34B—H34B	122.6
N10A—C33A—C38A	106.4 (5)	C34B—C35B—C36B	123.0 (6)
N10A—C33A—C34A	131.9 (7)	C34B—C35B—H35B	118.5
C38A—C33A—C34A	121.6 (6)	C36B—C35B—H35B	118.5
C35A—C34A—C33A	116.0 (7)	C37B—C36B—C35B	121.5 (6)
C35A—C34A—H34A	122.0	C37B—C36B—H36B	119.3
C33A—C34A—H34A	122.0	C35B—C36B—H36B	119.3
C34A—C35A—C36A	122.3 (7)	C36B—C37B—C38B	117.7 (6)
C34A—C35A—H35A	118.9	C36B—C37B—H37B	121.2
C36A—C35A—H35A	118.9	C38B—C37B—H37B	121.2
C37A—C36A—C35A	121.1 (7)	C33B—C38B—C37B	119.7 (6)
C37A—C36A—H36A	119.5	C33B—C38B—N9B	109.2 (5)
C35A—C36A—H36A	119.5	C37B—C38B—N9B	131.1 (6)
C36A—C37A—C38A	118.2 (7)	C15B—N1B—C7B	109.6 (4)
C36A—C37A—H37A	120.9	C15B—N1B—C1B	109.5 (4)
C38A—C37A—H37A	120.9	C7B—N1B—C1B	112.8 (4)
C37A—C38A—C33A	120.8 (6)	C15B—N1B—Pb2	106.2 (3)
C37A—C38A—N9A	130.3 (6)	C7B—N1B—Pb2	103.9 (3)
C33A—C38A—N9A	108.8 (5)	C1B—N1B—Pb2	114.5 (3)
C7A—N1A—C15A	109.7 (4)	C23B—N2B—C31B	110.3 (4)
C7A—N1A—C2A	112.5 (4)	C23B—N2B—C2B	112.0 (4)
C15A—N1A—C2A	108.0 (4)	C31B—N2B—C2B	108.7 (4)
C7A—N1A—Pb1	107.6 (3)	C23B—N2B—Pb2	106.9 (3)
C15A—N1A—Pb1	104.9 (3)	C31B—N2B—Pb2	103.5 (3)
C2A—N1A—Pb1	113.8 (3)	C2B—N2B—Pb2	115.0 (3)
C23A—N2A—C31A	109.0 (4)	C8B—N3B—C14B	104.4 (5)
C23A—N2A—C1A	110.5 (4)	C8B—N3B—Pb2	109.8 (3)
C31A—N2A—C1A	111.4 (4)	C14B—N3B—Pb2	139.7 (4)
C23A—N2A—Pb1	107.0 (3)	C8B—N4B—C9B	106.5 (5)
C31A—N2A—Pb1	105.7 (3)	C8B—N4B—H4B	126.7
C1A—N2A—Pb1	113.0 (3)	C9B—N4B—H4B	126.7
C8A—N3A—C14A	105.2 (5)	C16B—N5B—C22B	105.1 (5)
C8A—N3A—Pb1	112.2 (4)	C16B—N5B—Pb2	112.7 (4)
C14A—N3A—Pb1	141.9 (4)	C22B—N5B—Pb2	140.2 (4)
C8A—N4A—C9A	106.8 (5)	C16B—N6B—C17B	106.7 (5)
C8A—N4A—H4A	126.6	C16B—N6B—H6B	126.6
C9A—N4A—H4A	126.6	C17B—N6B—H6B	126.6
C16A—N5A—C22A	104.7 (5)	C24B—N7B—C30B	104.5 (5)
C16A—N5A—Pb1	112.4 (4)	C24B—N7B—Pb2	111.7 (4)
C22A—N5A—Pb1	142.7 (4)	C30B—N7B—Pb2	141.4 (4)
C16A—N6A—C17A	107.1 (5)	C24B—N8B—C25B	107.0 (5)
C16A—N6A—H6A	126.4	C24B—N8B—H8B	126.5
C17A—N6A—H6A	126.4	C25B—N8B—H8B	126.5
C24A—N7A—C30A	104.6 (5)	C32B—N9B—C38B	105.2 (5)
C24A—N7A—Pb1	112.4 (4)	C32B—N9B—Pb2	114.4 (4)
C30A—N7A—Pb1	141.2 (4)	C38B—N9B—Pb2	140.3 (4)
C24A—N8A—C25A	107.0 (5)	C32B—N10B—C33B	107.8 (5)
C24A—N8A—H8A	126.5	C32B—N10B—H10C	126.1
C25A—N8A—H8A	126.5	C33B—N10B—H10C	126.1
C32A—N9A—C38A	104.4 (5)	O2—N1—O1	119.1 (6)
C32A—N10A—C33A	106.5 (5)	O2—N1—O3	122.0 (6)
C32A—N10A—H10D	126.8	O1—N1—O3	118.8 (6)
C33A—N10A—H10D	126.8	O4—N2—O6	119.1 (6)
N9B—Pb2—N7B	100.93 (15)	O4—N2—O5	121.8 (5)
N9B—Pb2—N2B	66.41 (14)	O6—N2—O5	119.0 (6)
N7B—Pb2—N2B	64.53 (14)	O7—N3—O8	121.4 (9)
N9B—Pb2—N5B	142.51 (14)	O7—N3—O9	115.1 (9)
N7B—Pb2—N5B	80.04 (15)	O8—N3—O9	123.5 (8)
N2B—Pb2—N5B	81.07 (14)	O11—N4—O12	119.9 (8)
N9B—Pb2—N1B	86.72 (14)	O11—N4—O10	120.8 (10)
N7B—Pb2—N1B	120.68 (14)	O12—N4—O10	119.2 (10)
N2B—Pb2—N1B	65.68 (13)	H1O—O1A—H2O	113.6
N5B—Pb2—N1B	62.26 (14)	H3O—O13—H4O	137.6
N9B—Pb2—N3B	79.50 (14)	H5O—O14—H6O	108.0
N7B—Pb2—N3B	173.95 (14)		
			
N2A—C1A—C2A—N1A	−50.5 (6)	N1B—C7B—C8B—N3B	52.9 (8)
C6A—C1A—C2A—N1A	−178.9 (4)	N1B—C7B—C8B—N4B	−120.8 (6)
N2A—C1A—C2A—C3A	−178.1 (5)	N4B—C9B—C10B—C11B	175.8 (6)
C6A—C1A—C2A—C3A	53.4 (6)	C14B—C9B—C10B—C11B	−0.6 (10)
N1A—C2A—C3A—C4A	174.6 (5)	C9B—C10B—C11B—C12B	2.4 (10)
C1A—C2A—C3A—C4A	−57.1 (7)	C10B—C11B—C12B—C13B	−1.5 (11)
C2A—C3A—C4A—C5A	58.4 (7)	C11B—C12B—C13B—C14B	−1.3 (10)
C3A—C4A—C5A—C6A	−56.4 (7)	N4B—C9B—C14B—C13B	−179.3 (5)
N2A—C1A—C6A—C5A	178.5 (5)	C10B—C9B—C14B—C13B	−2.2 (9)
C2A—C1A—C6A—C5A	−53.2 (7)	N4B—C9B—C14B—N3B	0.0 (7)
C4A—C5A—C6A—C1A	55.1 (8)	C10B—C9B—C14B—N3B	177.1 (6)
N1A—C7A—C8A—N3A	−29.2 (8)	C12B—C13B—C14B—C9B	3.0 (9)
N1A—C7A—C8A—N4A	152.7 (5)	C12B—C13B—C14B—N3B	−176.1 (6)
N4A—C9A—C10A—C11A	179.4 (7)	N1B—C15B—C16B—N5B	27.4 (8)
C14A—C9A—C10A—C11A	−0.3 (11)	N1B—C15B—C16B—N6B	−151.6 (5)
C9A—C10A—C11A—C12A	−0.8 (12)	C22B—C17B—C18B—C19B	−1.2 (10)
C10A—C11A—C12A—C13A	0.4 (13)	N6B—C17B—C18B—C19B	178.1 (6)
C11A—C12A—C13A—C14A	1.1 (12)	C17B—C18B—C19B—C20B	0.9 (11)
N4A—C9A—C14A—N3A	0.9 (7)	C18B—C19B—C20B—C21B	−0.6 (12)
C10A—C9A—C14A—N3A	−179.4 (6)	C19B—C20B—C21B—C22B	0.5 (11)
N4A—C9A—C14A—C13A	−177.9 (6)	C18B—C17B—C22B—N5B	177.8 (6)
C10A—C9A—C14A—C13A	1.8 (11)	N6B—C17B—C22B—N5B	−1.7 (6)
C12A—C13A—C14A—C9A	−2.2 (10)	C18B—C17B—C22B—C21B	1.2 (9)
C12A—C13A—C14A—N3A	179.4 (7)	N6B—C17B—C22B—C21B	−178.3 (5)
N1A—C15A—C16A—N5A	−42.0 (7)	C20B—C21B—C22B—C17B	−0.8 (9)
N1A—C15A—C16A—N6A	139.3 (5)	C20B—C21B—C22B—N5B	−176.6 (6)
N6A—C17A—C18A—C19A	178.2 (6)	N2B—C23B—C24B—N7B	24.3 (9)
C22A—C17A—C18A—C19A	−0.4 (9)	N2B—C23B—C24B—N8B	−156.9 (5)
C17A—C18A—C19A—C20A	0.3 (9)	N8B—C25B—C26B—C27B	−179.0 (7)
C18A—C19A—C20A—C21A	−0.3 (10)	C30B—C25B—C26B—C27B	2.3 (11)
C19A—C20A—C21A—C22A	0.5 (10)	C25B—C26B—C27B—C28B	−1.4 (13)
N6A—C17A—C22A—N5A	−0.5 (6)	C26B—C27B—C28B—C29B	0.1 (13)
C18A—C17A—C22A—N5A	178.4 (5)	C27B—C28B—C29B—C30B	0.3 (12)
N6A—C17A—C22A—C21A	−178.3 (5)	N8B—C25B—C30B—N7B	−0.6 (7)
C18A—C17A—C22A—C21A	0.6 (9)	C26B—C25B—C30B—N7B	178.4 (6)
C20A—C21A—C22A—C17A	−0.6 (9)	N8B—C25B—C30B—C29B	179.1 (6)
C20A—C21A—C22A—N5A	−177.9 (6)	C26B—C25B—C30B—C29B	−1.9 (11)
N2A—C23A—C24A—N7A	−27.0 (8)	C28B—C29B—C30B—C25B	0.5 (10)
N2A—C23A—C24A—N8A	154.7 (5)	C28B—C29B—C30B—N7B	−179.8 (7)
N8A—C25A—C26A—C27A	−177.2 (6)	N2B—C31B—C32B—N9B	41.5 (7)
C30A—C25A—C26A—C27A	−1.3 (10)	N2B—C31B—C32B—N10B	−139.0 (5)
C25A—C26A—C27A—C28A	0.4 (11)	C38B—C33B—C34B—C35B	1.0 (9)
C26A—C27A—C28A—C29A	0.1 (12)	N10B—C33B—C34B—C35B	−178.3 (6)
C27A—C28A—C29A—C30A	0.3 (11)	C33B—C34B—C35B—C36B	0.0 (9)
C28A—C29A—C30A—C25A	−1.2 (10)	C34B—C35B—C36B—C37B	−0.6 (10)
C28A—C29A—C30A—N7A	177.1 (6)	C35B—C36B—C37B—C38B	0.2 (9)
N8A—C25A—C30A—C29A	178.6 (5)	N10B—C33B—C38B—C37B	178.0 (5)
C26A—C25A—C30A—C29A	1.8 (9)	C34B—C33B—C38B—C37B	−1.5 (9)
N8A—C25A—C30A—N7A	0.0 (6)	N10B—C33B—C38B—N9B	−0.1 (6)
C26A—C25A—C30A—N7A	−176.8 (6)	C34B—C33B—C38B—N9B	−179.6 (5)
N2A—C31A—C32A—N9A	−53.9 (8)	C36B—C37B—C38B—C33B	0.8 (9)
N2A—C31A—C32A—N10A	123.0 (6)	C36B—C37B—C38B—N9B	178.4 (6)
N10A—C33A—C34A—C35A	−176.5 (7)	C16B—C15B—N1B—C7B	−161.3 (5)
C38A—C33A—C34A—C35A	0.6 (10)	C16B—C15B—N1B—C1B	74.4 (6)
C33A—C34A—C35A—C36A	−1.9 (11)	C16B—C15B—N1B—Pb2	−49.7 (5)
C34A—C35A—C36A—C37A	0.8 (12)	C8B—C7B—N1B—C15B	58.9 (6)
C35A—C36A—C37A—C38A	1.6 (11)	C8B—C7B—N1B—C1B	−178.8 (5)
C36A—C37A—C38A—C33A	−2.9 (10)	C8B—C7B—N1B—Pb2	−54.3 (5)
C36A—C37A—C38A—N9A	175.1 (7)	C2B—C1B—N1B—C15B	−154.6 (5)
N10A—C33A—C38A—C37A	179.6 (6)	C6B—C1B—N1B—C15B	79.4 (6)
C34A—C33A—C38A—C37A	1.8 (10)	C2B—C1B—N1B—C7B	83.1 (6)
N10A—C33A—C38A—N9A	1.2 (7)	C6B—C1B—N1B—C7B	−43.0 (6)
C34A—C33A—C38A—N9A	−176.6 (6)	C2B—C1B—N1B—Pb2	−35.4 (6)
C8A—C7A—N1A—C15A	−67.3 (6)	C6B—C1B—N1B—Pb2	−161.5 (4)
C8A—C7A—N1A—C2A	172.3 (5)	N9B—Pb2—N1B—C15B	−162.4 (3)
C8A—C7A—N1A—Pb1	46.2 (5)	N7B—Pb2—N1B—C15B	96.8 (3)
C16A—C15A—N1A—C7A	166.4 (5)	N2B—Pb2—N1B—C15B	132.0 (3)
C16A—C15A—N1A—C2A	−70.6 (5)	N5B—Pb2—N1B—C15B	39.4 (3)
C16A—C15A—N1A—Pb1	51.2 (5)	N3B—Pb2—N1B—C15B	−82.5 (3)
C1A—C2A—N1A—C7A	−84.0 (6)	N9B—Pb2—N1B—C7B	−46.9 (3)
C3A—C2A—N1A—C7A	42.7 (6)	N7B—Pb2—N1B—C7B	−147.6 (3)
C1A—C2A—N1A—C15A	154.6 (4)	N2B—Pb2—N1B—C7B	−112.4 (3)
C3A—C2A—N1A—C15A	−78.6 (6)	N5B—Pb2—N1B—C7B	155.0 (4)
C1A—C2A—N1A—Pb1	38.6 (5)	N3B—Pb2—N1B—C7B	33.1 (3)
C3A—C2A—N1A—Pb1	165.3 (4)	N9B—Pb2—N1B—C1B	76.6 (4)
N5A—Pb1—N1A—C7A	−152.0 (4)	N7B—Pb2—N1B—C1B	−24.2 (4)
N3A—Pb1—N1A—C7A	−35.2 (3)	N2B—Pb2—N1B—C1B	11.0 (3)
N7A—Pb1—N1A—C7A	47.1 (3)	N5B—Pb2—N1B—C1B	−81.6 (4)
N2A—Pb1—N1A—C7A	111.6 (3)	N3B—Pb2—N1B—C1B	156.5 (4)
N5A—Pb1—N1A—C15A	−35.2 (3)	C24B—C23B—N2B—C31B	67.1 (6)
N3A—Pb1—N1A—C15A	81.6 (3)	C24B—C23B—N2B—C2B	−171.7 (5)
N7A—Pb1—N1A—C15A	163.9 (3)	C24B—C23B—N2B—Pb2	−44.9 (5)
N2A—Pb1—N1A—C15A	−131.6 (3)	C32B—C31B—N2B—C23B	−164.5 (5)
N5A—Pb1—N1A—C2A	82.6 (3)	C32B—C31B—N2B—C2B	72.3 (5)
N3A—Pb1—N1A—C2A	−160.6 (4)	C32B—C31B—N2B—Pb2	−50.4 (5)
N7A—Pb1—N1A—C2A	−78.2 (3)	C1B—C2B—N2B—C23B	83.2 (6)
N2A—Pb1—N1A—C2A	−13.8 (3)	C3B—C2B—N2B—C23B	−43.4 (6)
C24A—C23A—N2A—C31A	161.7 (5)	C1B—C2B—N2B—C31B	−154.7 (4)
C24A—C23A—N2A—C1A	−75.5 (6)	C3B—C2B—N2B—C31B	78.7 (6)
C24A—C23A—N2A—Pb1	47.9 (5)	C1B—C2B—N2B—Pb2	−39.2 (5)
C32A—C31A—N2A—C23A	−61.7 (6)	C3B—C2B—N2B—Pb2	−165.8 (4)
C32A—C31A—N2A—C1A	176.2 (5)	N9B—Pb2—N2B—C23B	152.0 (4)
C32A—C31A—N2A—Pb1	53.1 (5)	N7B—Pb2—N2B—C23B	36.1 (3)
C2A—C1A—N2A—C23A	155.7 (5)	N5B—Pb2—N2B—C23B	−47.1 (3)
C6A—C1A—N2A—C23A	−77.4 (6)	N1B—Pb2—N2B—C23B	−110.6 (4)
C2A—C1A—N2A—C31A	−82.9 (6)	N3B—Pb2—N2B—C23B	−147.4 (3)
C6A—C1A—N2A—C31A	44.0 (6)	N9B—Pb2—N2B—C31B	35.5 (3)
C2A—C1A—N2A—Pb1	35.9 (6)	N7B—Pb2—N2B—C31B	−80.4 (3)
C6A—C1A—N2A—Pb1	162.8 (4)	N5B—Pb2—N2B—C31B	−163.5 (3)
N5A—Pb1—N2A—C23A	160.7 (3)	N1B—Pb2—N2B—C31B	132.9 (3)
N1A—Pb1—N2A—C23A	−133.2 (3)	N3B—Pb2—N2B—C31B	96.2 (3)
N3A—Pb1—N2A—C23A	−96.4 (3)	N9B—Pb2—N2B—C2B	−82.9 (4)
N7A—Pb1—N2A—C23A	−38.6 (3)	N7B—Pb2—N2B—C2B	161.1 (4)
N5A—Pb1—N2A—C31A	44.6 (3)	N5B—Pb2—N2B—C2B	78.0 (4)
N1A—Pb1—N2A—C31A	110.7 (3)	N1B—Pb2—N2B—C2B	14.5 (3)
N3A—Pb1—N2A—C31A	147.4 (3)	N3B—Pb2—N2B—C2B	−22.3 (4)
N7A—Pb1—N2A—C31A	−154.8 (4)	N4B—C8B—N3B—C14B	−0.5 (7)
N5A—Pb1—N2A—C1A	−77.5 (4)	C7B—C8B—N3B—C14B	−174.7 (5)
N1A—Pb1—N2A—C1A	−11.4 (3)	N4B—C8B—N3B—Pb2	158.0 (4)
N3A—Pb1—N2A—C1A	25.3 (4)	C7B—C8B—N3B—Pb2	−16.2 (7)
N7A—Pb1—N2A—C1A	83.1 (4)	C9B—C14B—N3B—C8B	0.3 (7)
N4A—C8A—N3A—C14A	−0.1 (7)	C13B—C14B—N3B—C8B	179.5 (6)
C7A—C8A—N3A—C14A	−178.4 (6)	C9B—C14B—N3B—Pb2	−147.4 (5)
N4A—C8A—N3A—Pb1	172.5 (4)	C13B—C14B—N3B—Pb2	31.8 (10)
C7A—C8A—N3A—Pb1	−5.7 (8)	N9B—Pb2—N3B—C8B	80.7 (4)
C9A—C14A—N3A—C8A	−0.5 (7)	N2B—Pb2—N3B—C8B	26.4 (4)
C13A—C14A—N3A—C8A	178.1 (7)	N5B—Pb2—N3B—C8B	−61.1 (4)
C9A—C14A—N3A—Pb1	−169.4 (5)	N1B—Pb2—N3B—C8B	−10.5 (4)
C13A—C14A—N3A—Pb1	9.2 (12)	N9B—Pb2—N3B—C14B	−132.7 (6)
N5A—Pb1—N3A—C8A	79.2 (4)	N2B—Pb2—N3B—C14B	173.0 (5)
N1A—Pb1—N3A—C8A	22.1 (4)	N5B—Pb2—N3B—C14B	85.6 (6)
N7A—Pb1—N3A—C8A	−66.0 (4)	N1B—Pb2—N3B—C14B	136.1 (6)
N2A—Pb1—N3A—C8A	−15.4 (5)	N3B—C8B—N4B—C9B	0.5 (7)
N5A—Pb1—N3A—C14A	−112.4 (7)	C7B—C8B—N4B—C9B	174.8 (5)
N1A—Pb1—N3A—C14A	−169.5 (7)	C14B—C9B—N4B—C8B	−0.3 (6)
N7A—Pb1—N3A—C14A	102.5 (7)	C10B—C9B—N4B—C8B	−177.1 (7)
N2A—Pb1—N3A—C14A	153.0 (6)	N6B—C16B—N5B—C22B	−1.0 (6)
N3A—C8A—N4A—C9A	0.7 (7)	C15B—C16B—N5B—C22B	179.9 (5)
C7A—C8A—N4A—C9A	179.0 (6)	N6B—C16B—N5B—Pb2	−168.4 (4)
C14A—C9A—N4A—C8A	−0.9 (7)	C15B—C16B—N5B—Pb2	12.5 (7)
C10A—C9A—N4A—C8A	179.4 (7)	C17B—C22B—N5B—C16B	1.6 (6)
N6A—C16A—N5A—C22A	1.3 (6)	C21B—C22B—N5B—C16B	177.8 (6)
C15A—C16A—N5A—C22A	−177.5 (5)	C17B—C22B—N5B—Pb2	163.4 (4)
N6A—C16A—N5A—Pb1	−175.1 (3)	C21B—C22B—N5B—Pb2	−20.5 (10)
C15A—C16A—N5A—Pb1	6.0 (7)	N9B—Pb2—N5B—C16B	−65.3 (5)
C17A—C22A—N5A—C16A	−0.4 (6)	N7B—Pb2—N5B—C16B	−160.3 (4)
C21A—C22A—N5A—C16A	177.0 (6)	N2B—Pb2—N5B—C16B	−94.8 (4)
C17A—C22A—N5A—Pb1	174.1 (4)	N1B—Pb2—N5B—C16B	−27.7 (4)
C21A—C22A—N5A—Pb1	−8.4 (11)	N3B—Pb2—N5B—C16B	24.8 (4)
N1A—Pb1—N5A—C16A	16.7 (4)	N9B—Pb2—N5B—C22B	133.8 (5)
N3A—Pb1—N5A—C16A	−39.6 (4)	N7B—Pb2—N5B—C22B	38.8 (6)
N7A—Pb1—N5A—C16A	52.1 (5)	N2B—Pb2—N5B—C22B	104.3 (6)
N2A—Pb1—N5A—C16A	84.1 (4)	N1B—Pb2—N5B—C22B	171.5 (6)
N1A—Pb1—N5A—C22A	−157.5 (6)	N3B—Pb2—N5B—C22B	−136.1 (6)
N3A—Pb1—N5A—C22A	146.1 (6)	N5B—C16B—N6B—C17B	0.0 (7)
N7A—Pb1—N5A—C22A	−122.2 (6)	C15B—C16B—N6B—C17B	179.1 (5)
N2A—Pb1—N5A—C22A	−90.2 (6)	C18B—C17B—N6B—C16B	−178.4 (7)
N5A—C16A—N6A—C17A	−1.7 (6)	C22B—C17B—N6B—C16B	1.1 (6)
C15A—C16A—N6A—C17A	177.2 (5)	N8B—C24B—N7B—C30B	−0.4 (7)
C22A—C17A—N6A—C16A	1.3 (6)	C23B—C24B—N7B—C30B	178.4 (6)
C18A—C17A—N6A—C16A	−177.5 (6)	N8B—C24B—N7B—Pb2	−166.9 (4)
N8A—C24A—N7A—C30A	−0.5 (6)	C23B—C24B—N7B—Pb2	12.0 (8)
C23A—C24A—N7A—C30A	−178.9 (5)	C25B—C30B—N7B—C24B	0.6 (7)
N8A—C24A—N7A—Pb1	167.4 (4)	C29B—C30B—N7B—C24B	−179.1 (7)
C23A—C24A—N7A—Pb1	−10.9 (7)	C25B—C30B—N7B—Pb2	160.2 (5)
C29A—C30A—N7A—C24A	−178.1 (6)	C29B—C30B—N7B—Pb2	−19.5 (12)
C25A—C30A—N7A—C24A	0.3 (6)	N9B—Pb2—N7B—C24B	−82.5 (4)
C29A—C30A—N7A—Pb1	19.8 (11)	N2B—Pb2—N7B—C24B	−25.4 (4)
C25A—C30A—N7A—Pb1	−161.8 (4)	N5B—Pb2—N7B—C24B	59.3 (4)
N5A—Pb1—N7A—C24A	62.0 (5)	N1B—Pb2—N7B—C24B	10.2 (5)
N1A—Pb1—N7A—C24A	94.3 (4)	N9B—Pb2—N7B—C30B	118.8 (6)
N3A—Pb1—N7A—C24A	160.5 (4)	N2B—Pb2—N7B—C30B	175.9 (7)
N2A—Pb1—N7A—C24A	26.1 (4)	N5B—Pb2—N7B—C30B	−99.3 (6)
N5A—Pb1—N7A—C30A	−136.7 (6)	N1B—Pb2—N7B—C30B	−148.5 (6)
N1A—Pb1—N7A—C30A	−104.4 (6)	N7B—C24B—N8B—C25B	0.1 (7)
N3A—Pb1—N7A—C30A	−38.3 (6)	C23B—C24B—N8B—C25B	−178.9 (6)
N2A—Pb1—N7A—C30A	−172.7 (6)	C30B—C25B—N8B—C24B	0.3 (7)
N7A—C24A—N8A—C25A	0.5 (7)	C26B—C25B—N8B—C24B	−178.5 (7)
C23A—C24A—N8A—C25A	179.0 (5)	N10B—C32B—N9B—C38B	−1.0 (6)
C30A—C25A—N8A—C24A	−0.3 (6)	C31B—C32B—N9B—C38B	178.6 (5)
C26A—C25A—N8A—C24A	176.1 (7)	N10B—C32B—N9B—Pb2	176.0 (3)
N10A—C32A—N9A—C38A	1.1 (7)	C31B—C32B—N9B—Pb2	−4.4 (7)
C31A—C32A—N9A—C38A	178.2 (6)	C33B—C38B—N9B—C32B	0.6 (6)
C37A—C38A—N9A—C32A	−179.5 (7)	C37B—C38B—N9B—C32B	−177.2 (6)
C33A—C38A—N9A—C32A	−1.4 (7)	C33B—C38B—N9B—Pb2	−175.0 (4)
N9A—C32A—N10A—C33A	−0.3 (7)	C37B—C38B—N9B—Pb2	7.1 (10)
C31A—C32A—N10A—C33A	−177.6 (5)	N7B—Pb2—N9B—C32B	38.2 (4)
C38A—C33A—N10A—C32A	−0.5 (7)	N2B—Pb2—N9B—C32B	−17.6 (4)
C34A—C33A—N10A—C32A	176.9 (7)	N5B—Pb2—N9B—C32B	−49.7 (5)
N1B—C1B—C2B—N2B	50.1 (6)	N1B—Pb2—N9B—C32B	−82.5 (4)
C6B—C1B—C2B—N2B	178.5 (4)	N3B—Pb2—N9B—C32B	−148.0 (4)
N1B—C1B—C2B—C3B	177.8 (5)	N7B—Pb2—N9B—C38B	−146.4 (5)
C6B—C1B—C2B—C3B	−53.8 (6)	N2B—Pb2—N9B—C38B	157.8 (6)
N2B—C2B—C3B—C4B	−174.6 (5)	N5B—Pb2—N9B—C38B	125.7 (5)
C1B—C2B—C3B—C4B	57.2 (7)	N1B—Pb2—N9B—C38B	92.9 (6)
C2B—C3B—C4B—C5B	−58.3 (7)	N3B—Pb2—N9B—C38B	27.4 (5)
C3B—C4B—C5B—C6B	56.4 (7)	N9B—C32B—N10B—C33B	0.9 (6)
C4B—C5B—C6B—C1B	−56.0 (8)	C31B—C32B—N10B—C33B	−178.7 (5)
N1B—C1B—C6B—C5B	−177.5 (5)	C38B—C33B—N10B—C32B	−0.5 (6)
C2B—C1B—C6B—C5B	54.6 (7)	C34B—C33B—N10B—C32B	179.0 (6)

### Hydrogen-bond geometry (Å, °)

**Table:**

*D*—H···*A*	*D*—H	H···*A*	*D*···*A*	*D*—H···*A*
N4A—H4A···O9^i^	0.86	2.14	2.926 (8)	151.
N4A—H4A···O7^i^	0.86	2.45	3.242 (10)	153.
N6A—H6A···O3	0.86	2.04	2.848 (7)	157.
N8A—H8A···O11^ii^	0.86	2.12	2.898 (8)	150.
N8A—H8A···O12^ii^	0.86	2.37	3.091 (8)	141.
N10A—H10D···O2^iii^	0.86	2.03	2.890 (7)	175.
N4B—H4B···O4^iv^	0.86	2.03	2.879 (6)	169.
N4B—H4B···O6^iv^	0.86	2.57	3.256 (7)	138.
N6B—H6B···O14	0.86	1.95	2.784 (6)	164.
N8B—H8B···O13^v^	0.86	1.99	2.843 (6)	169.
N10B—H10C···O5	0.86	2.06	2.852 (6)	152.
O1A—H1O···O13	0.84	2.13	2.646 (13)	120.
O1A—H2O···O10	0.84	1.79	2.630 (15)	178.
O13—H3O···O10	0.84	2.46	3.126 (9)	137.
O13—H4O···O1^v^	0.84	2.31	3.029 (8)	143.
O14—H5O···O9^vi^	0.84	2.01	2.853 (8)	179.
O14—H6O···O12^vii^	0.84	2.10	2.939 (8)	179.
C13B—H13B···O7	0.93	2.50	3.312 (10)	146.
C15A—H15A···O4	0.97	2.59	3.447 (7)	148.
C18B—H18B···O10^vii^	0.93	2.45	3.377 (9)	171.
C7B—H7B1···O6^iv^	0.97	2.31	3.227 (8)	157.
C29B—H29B···O8	0.93	2.57	3.363 (10)	144.
C31A—H31B···O1^iii^	0.97	2.41	3.313 (8)	155.

Symmetry codes: (i) −*x*, −*y*+1, −*z*+1; (ii) *x*−1, *y*, *z*; (iii) −*x*, −*y*+1, −*z*; (iv) −*x*+1, −*y*+1, −*z*+1; (v) −*x*+1, −*y*+1, −*z*; (vi) *x*+1, *y*, *z*; (vii) *x*, *y*+1, *z*.
